# Transcranial magnetic stimulation (TMS) protocol on lower limb muscle strength in healthy individuals

**DOI:** 10.1016/j.mex.2025.103335

**Published:** 2025-04-25

**Authors:** Thiago Conceição dos Santos, Rildo Tavares dos Santos, Hudson Renatode Paula Oliveira, Felipe Mendes Barcelos Angeli, Emanuelly Augustada Silva Bispo, Ian Manhoni Bahiense, Walter Gomesda Silva Filho, Guilherme Peixoto Tinoco Areas, Fernando Zanelada Silva Arêas

**Affiliations:** a Neurorehabilitation and Neuromodulation Laboratory, Department of Physiological Sciences, Federal University of Espírito Santo, City of Vitória, ES, Brazil; b Physical Education and Sports Center, Federal University of Espírito Santo, Espírito Santo, Brasil; c Postgraduate Progam in Human Movement Sciences. Federal University of Amazonas, Manaus, AM, Brazil; d Physiology Science Laboratory, Federal University of Amazonas, Manaus, AM, Brazil; e Postgraduate program in Physiological Sciences, Federal University of Espírito Santo, City of Vitória, ES, Brazil; f Baylor Scott and White Research Institute and Baylor Scott and White Institute for Rehabilitation, Dallas, TX. USA

**Keywords:** Muscle strength, Neuromodulation, Transcranial magnetic stimulation, Isokinetic dynamometer, Isokinetic, Protocol

## Abstract

This study will investigate the effects of high-frequency Transcranial Magnetic Stimulation (TMS) on lower limb muscle strength, using an isokinetic dynamometer to measure strength changes. The research will involve 50 healthy sedentary male participants aged 18–25, randomized into active and sham groups. The protocol will integrate TMS with surface electromyography (sEMG) to assess muscle activation and enhance accuracy. The aim will be to explore whether TMS can influence peripheral neuromuscular characteristics, improving muscle performance.

The protocol will involve a double-blind, randomized design and the use of an isokinetic dynamometer for precise muscle strength measurement. By employing TMS, which enhances cortical excitability, and evaluating muscle performance via controlled assessments, the study will seek to provide reliable pre- and post-intervention data. The study’s sample calculation will ensure adequate statistical power and account for potential attrition, with 50 participants included.

This research will have potential clinical applications, particularly for muscle rehabilitation and performance optimization. However, it will face limitations, such as a male-only sample and variability in individual responses to TMS. Despite these challenges, the study's findings could inform future therapeutic strategies for enhancing muscle strength and contribute to advancing TMS applications in both clinical and athletic settings.

## Graphical abstract

**Figure:**
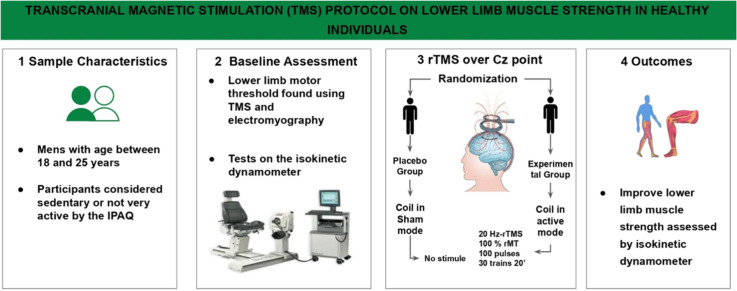

Specifications table

**Table:** 

Subject area:	Neuroscience
More specific subject area:	Noninvasive Neuromodulation
Name of your protocol:	TMS on force lower limb muscles of healthy individuals
Reagents/tools:	TMS MagPro R30 Biodex System Pro isokinetic dynamometer New Miotool
Experimental design:	Randomized clinical trial with sedentary individuals, comparing placebo and EMT intervention, assessing muscle strength before and after stimulation.
Trial registration:	Informed consent was obtained from the participants.
Ethics:	Informed consent was obtained from all patients after patient eligibility was confirmed. The entire research project was submitted to the Research Ethics Committee of the Federal University of Espírito Santo, and approval was obtained in accordance with substantiated opinion number CAAE80859824200005060.
Value of the Protocol:	The protocol aims to evaluate the potential of improving muscle strength through transcranial magnetic stimulation

## Background

Skeletal muscles exhibit four primary performance characteristics: (1) maximum force production, (2) contraction velocity, (3) maximum power generation, and (4) contraction efficiency. Maximum force production is dictated by the amount of myosin and actin, as force is generated through cross-bridges formed by myosin binding to actin, which directly correlates with the muscle fiber cross-sectional area.

Contraction velocity refers to the maximum speed at which a muscle fiber can shorten, a property dependent on the velocity of the cross-bridge cycle. Maximum power generation is defined as the product of force production and shortening velocity (force × velocity). Lastly, contraction efficiency quantifies the energy expenditure relative to the force generated, calculated as the ratio of energy consumed to force output [1].

Each motor neuron originating from the spinal cord innervates multiple muscle fibers, with the number of fibers varying according to muscle type. All muscle fibers innervated by a single nerve fiber constitute a motor unit. When the central nervous system (CNS) sends a weak signal for muscle contraction, smaller motor units are preferentially recruited over larger ones. As CNS signal strength increases, larger motor units are activated, with some capable of producing up to 50 times the contractile force of smaller units. This recruitment pattern, known as the size principle, allows fine gradation of force during weak contractions while ensuring progressive force generation when needed [2].

At low stimulation frequencies, skeletal muscle contractions occur sequentially. As frequency increases, contractions begin before the previous ones have fully relaxed, summating to enhance total contractile force. Beyond a critical frequency, contractions fuse into a smooth, continuous contraction. At slightly higher frequencies, maximal force production is reached, beyond which further increases in frequency no longer enhance contractile force [2].

Transcranial magnetic stimulation (TMS) is a non-invasive brain stimulation technique [3]. High-frequency TMS (>5 Hz) has been shown to enhance cortical excitability, particularly within the motor cortex [4].

Muscle strength can be assessed using various methods, including maximum weight lifted with exercise equipment, maximum isometric torque, and maximum isokinetic torque at specific or nonspecific angles. The isokinetic dynamometer enables precise evaluation of maximum concentric and eccentric muscle strength at constant velocities over a controlled range of motion [5].

Since TMS, when applied to the CNS, has the potential to influence the characteristics of the peripheral neuromuscular system, this study aims to investigate whether TMS can enhance muscle performance. The isokinetic dynamometer is used as a reference tool to ensure reliable pre- and post-intervention measurements, facilitating reproducibility in future studies and clinical applications.

## Description of protocol

This study aims to evaluate the effects of electromagnetic stimulation on lower limb muscle strength, using an isokinetic dynamometer for assessment. Surface electromyography (sEMG) is employed to determine the intensity of TMS by capturing muscle activation at baseline, thereby enhancing the protocol's accuracy.

**Fig. 1:**
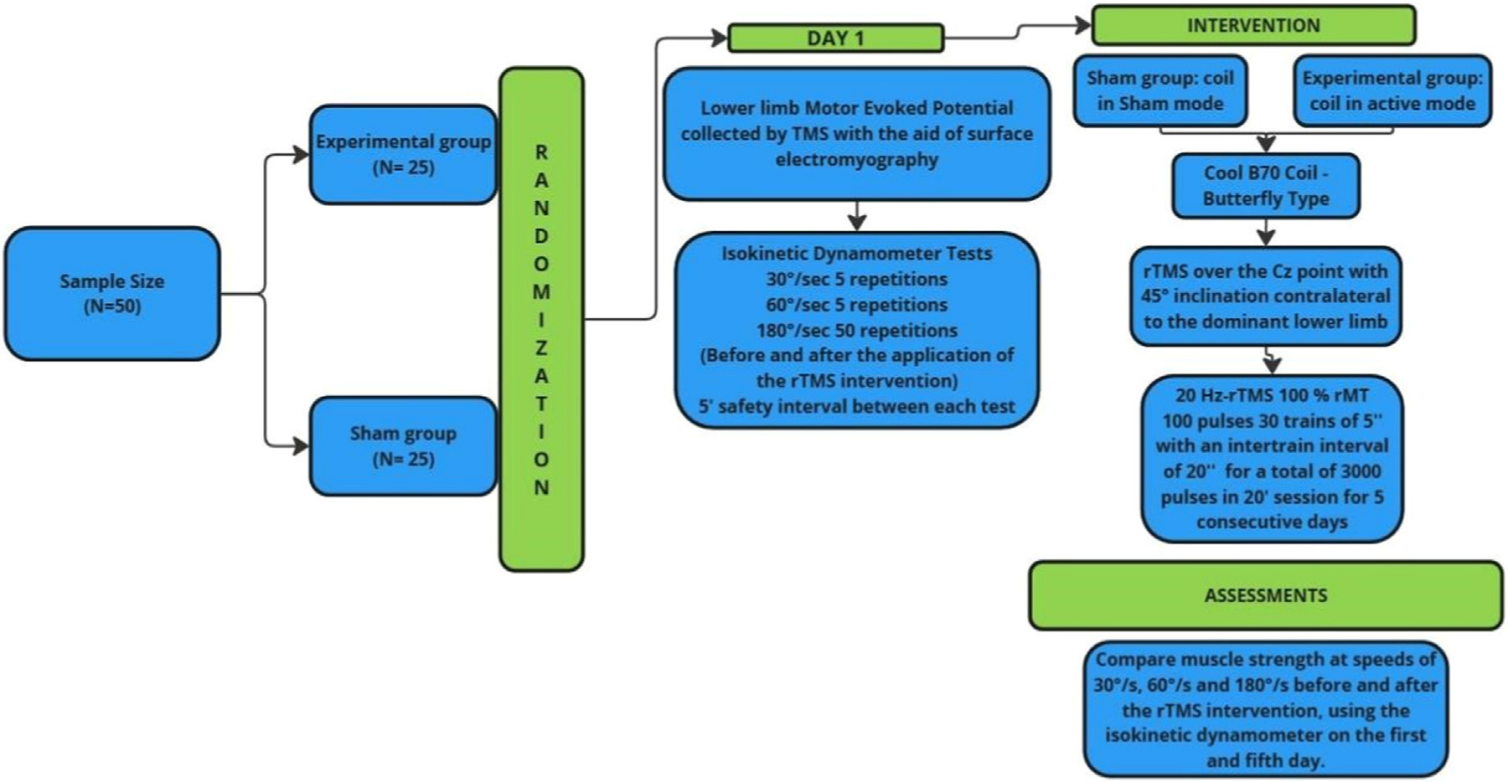
Study flowchart.

This is a double-blind, randomized clinical trial conducted at the School Clinic of the Federal University of Espírito Santo (UFES). The study includes 50 healthy participants, aged 18 to 25, classified as sedentary according to the International Physical Activity Questionnaire (IPAQ) and meeting the inclusion criteria (Fig. 1).

## Sample calculation

To date, no studies have assessed the effects of TMS on large muscle groups, such as the quadriceps, using an isokinetic approach. Additionally, no established clinically significant difference exists for isokinetic quadriceps strength.

For this study, the effect size (Cohen’s f) for the statistical calculation of a two-way (between-within) ANOVA with two groups and two time points was set at 0.20. The following parameters were applied: effect size = 0.20, alpha = 0.05, statistical power = 80 %, number of groups = 2, number of measurements = 2, correlation between repeated measures = 0.5, and non-sphericity correction = 1. Based on these parameters, 42 volunteers were required. Considering a potential 20 % sample attrition, the final sample size was adjusted to 50 participants, with 25 allocated to the sham group and 25 to the intervention group (Table 1).

**Table 1:** Inclusion and exclusion criteria study.

Inclusion criteria	Exclusion criteria
1. Men aged between 18 and 50 years.	1. Have had deep brain stimulation (DBS) surgery.
2. Identified as sedentary or minimally active according to the IPAQ questionnaire.	2. Presence of associated neurological diseases.
3. Physically capable of performing isokinetic dynamometer tests.	3. Orthopedic conditions that limit participation in study.
4. Be able to understand instructions for the isokinetic dynamometer tests.	4. History of seizures or traumatic brain injury.
5. Scoring adequately on the Mini-Mental State Examination, adjusted for educational level (26 for higher education, 18 for primary and secondary education, 13 for illiterates).	5. Acute strain (musculotendinous unit) or sprain (non-contractile tissue).
6. Able to give informed consent in accordance with institutional policies.	6. Soft tissue healing restrictions (e.g., post-surgery).
7. Capable of completing all physical and cognitive test requirements as defined.	7. Pain during protocol execution.
	8. Extremely limited range of motion (ROM).
	9. Severe effusion.
	10 Joint instability.
	11. Subacute or chronic third-degree sprains.
	12. Significant knee ROM limitation.
	13. Lower limb joint laxity.

## Materials and methods

Participant allocation will be concealed using sequentially numbered, opaque, sealed envelopes, prepared prior to the study by a research assistant who will not be involved in data collection or analysis. After obtaining baseline measurements, participants will be randomly assigned to either the experimental or control group. A separate research assistant will be responsible for setting the stimulator to active TMS or placebo, ensuring that the therapists administering the intervention remain blinded to group allocation. All outcome assessments will be conducted by blinded evaluators.

Participants will be allocated to either the active stimulation or placebo (sham) group. Both groups will undergo TMS to collect motor-evoked potential (MEP) data, which will be used to calibrate individual stimulation parameters. This procedure will be supported by electromyography (EMG) to ensure data accuracy.

On the first day of testing, following MEP data collection, all participants will undergo a familiarization period with the isokinetic dynamometer. During this session, they will perform practice tests to become acquainted with the equipment, followed by isokinetic assessments at speeds of 30°/s and 60°/s, with five repetitions at each speed. After a five-minute rest period, an additional test will be conducted before and after TMS application, consisting of 50 repetitions at a speed of 180°/s. To assess the coefficient of variation during testing, participants will not be informed of the number of repetitions in advance. This procedure will be repeated on both the first and fifth days of stimulation.

In the active group, TMS will be delivered using a Cool B70 butterfly coil (Magventure, Denmark) positioned over the primary motor cortex. Stimulation intensity will be set at 100 % of the motor threshold, defined as the lowest intensity capable of inducing peripheral muscle contraction, verified via EMG. The stimulation protocol will follow the 10/20 EEG system, with Cz serving as the common stimulation site for all participants. Coil orientation will depend on the dominant limb: for right-leg-dominant participants, the coil will be tilted 45° to the left, while for left-leg-dominant participants, it will be tilted 45° to the right. The protocol will consist of 30 trains of 100 pulses at 20 Hz, with each train lasting 5 s and separated by a 20-second inter-train interval, totaling 3000 pulses over approximately 20 min. This procedure will be repeated for five consecutive days, with individual motor threshold reassessed daily to ensure accurate stimulation parameters.

The placebo group will receive sham stimulation using a Cool B70 butterfly coil (Magventure, Denmark) designed to replicate the sound and timing of active stimulation without delivering a magnetic current.

On the fifth day, following stimulation, both groups will rest for five minutes before performing the same isokinetic dynamometer test as on the first day. The collected data will be compared with pre-stimulation results.

The study will be conducted at the School Clinic of the Federal University of Espírito Santo (UFES). All team members will receive standardized training on the use of the isokinetic dynamometer and TMS equipment. The TMS operators will remain blinded to group allocation, as the stimulator will be pre-configured by an external researcher with no direct contact with participants or investigators. During both isokinetic tests, participants will be instructed to exert maximum effort throughout all repetitions, with auditory stimuli provided to maintain engagement during testing.

Participants unable to complete the test will be classified as "incomplete tests," and a replacement participant will be recruited.

### International Physical Activity Questionnaire (IPAQ)

**Fig. 2:**
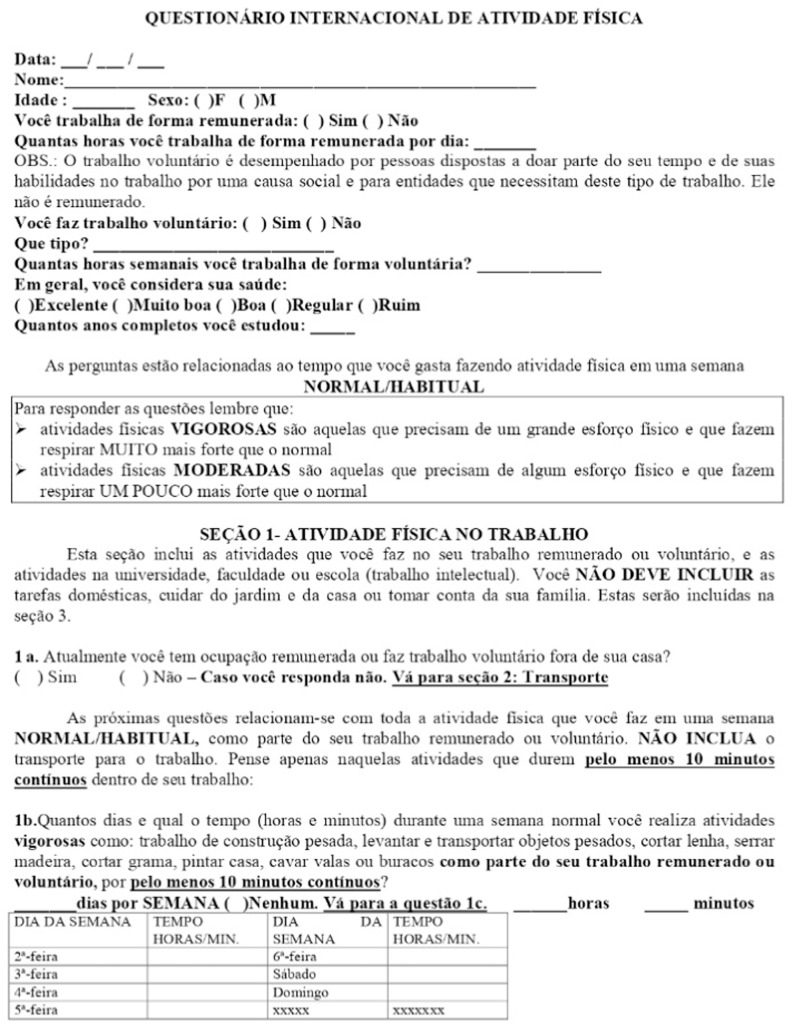

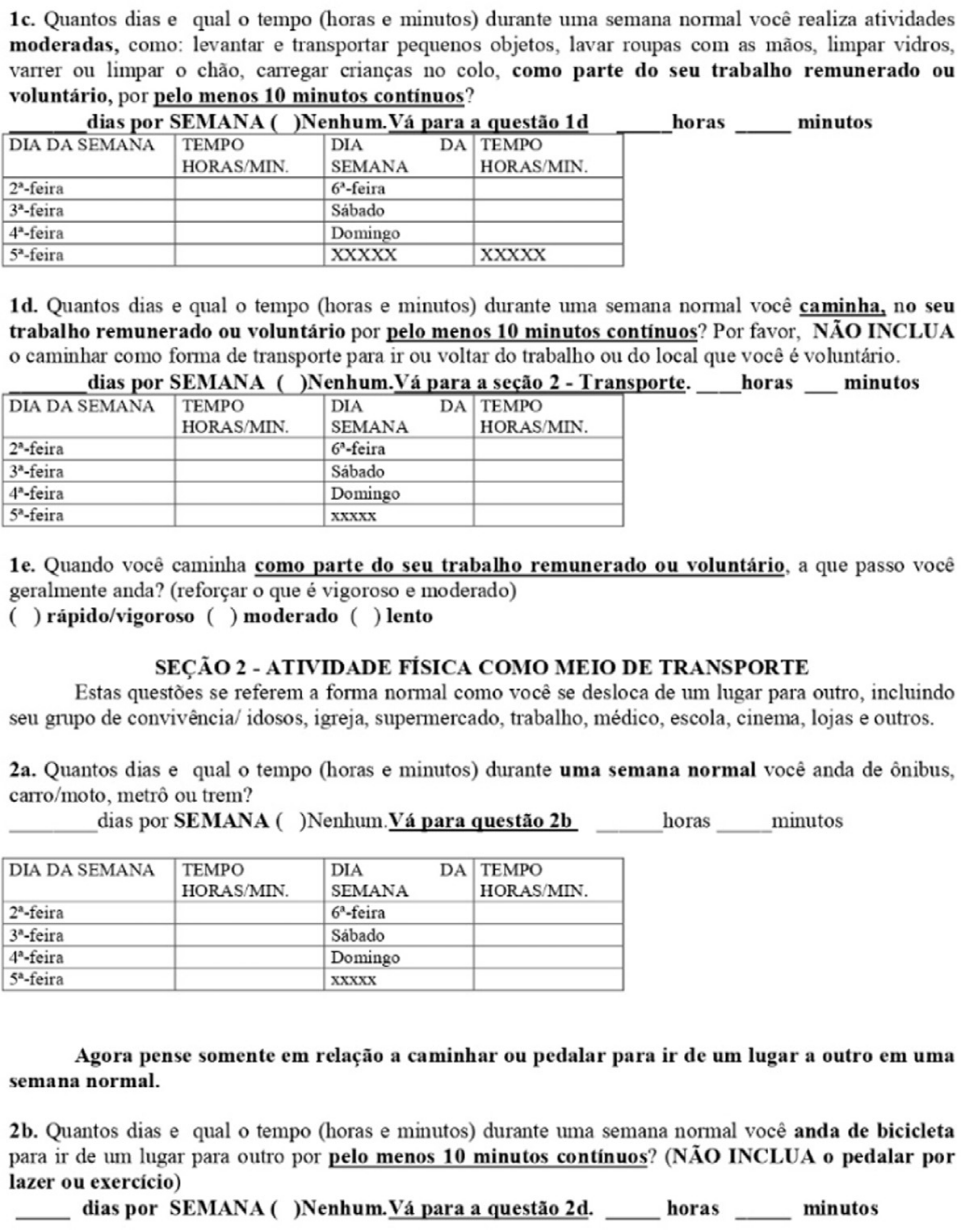

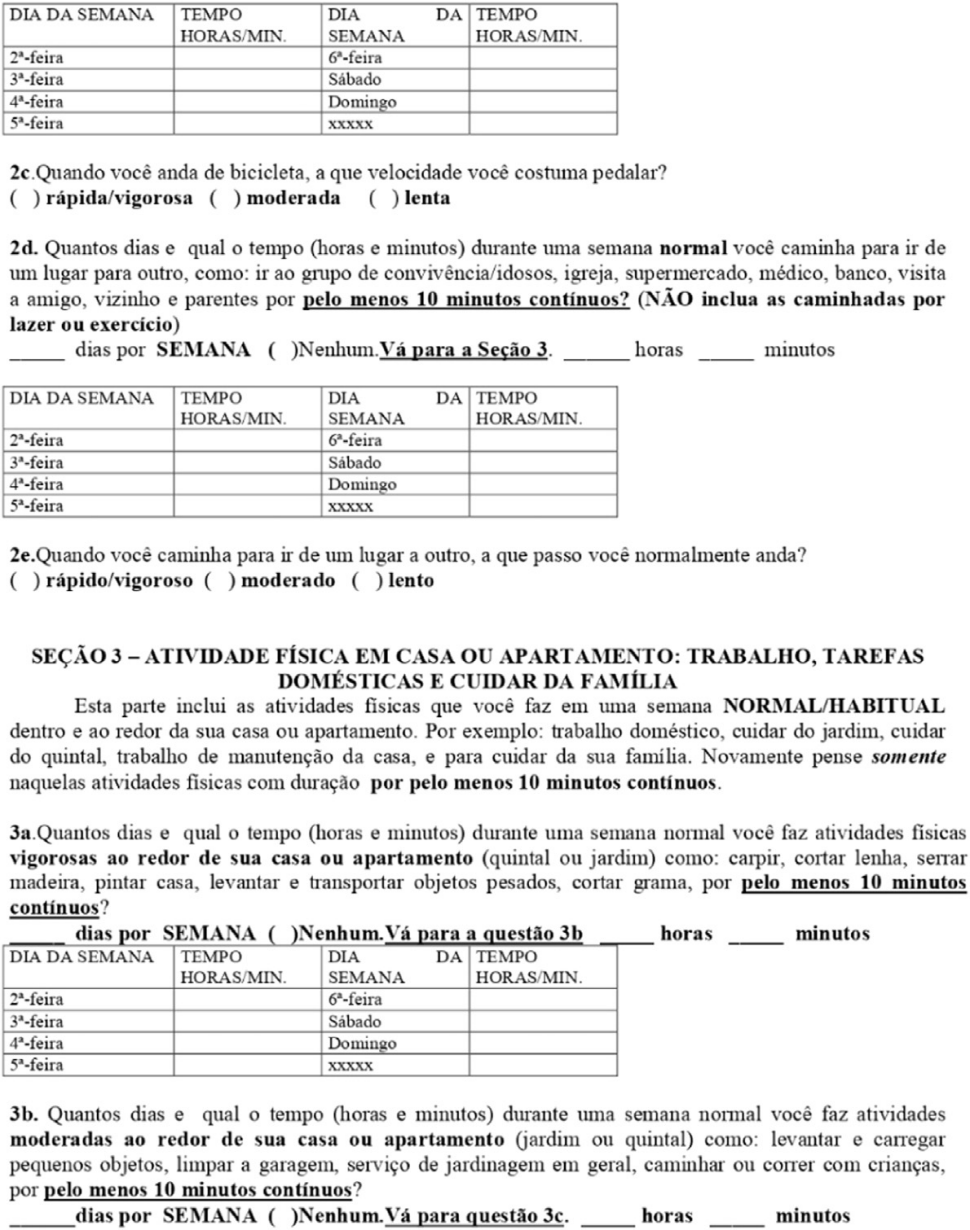

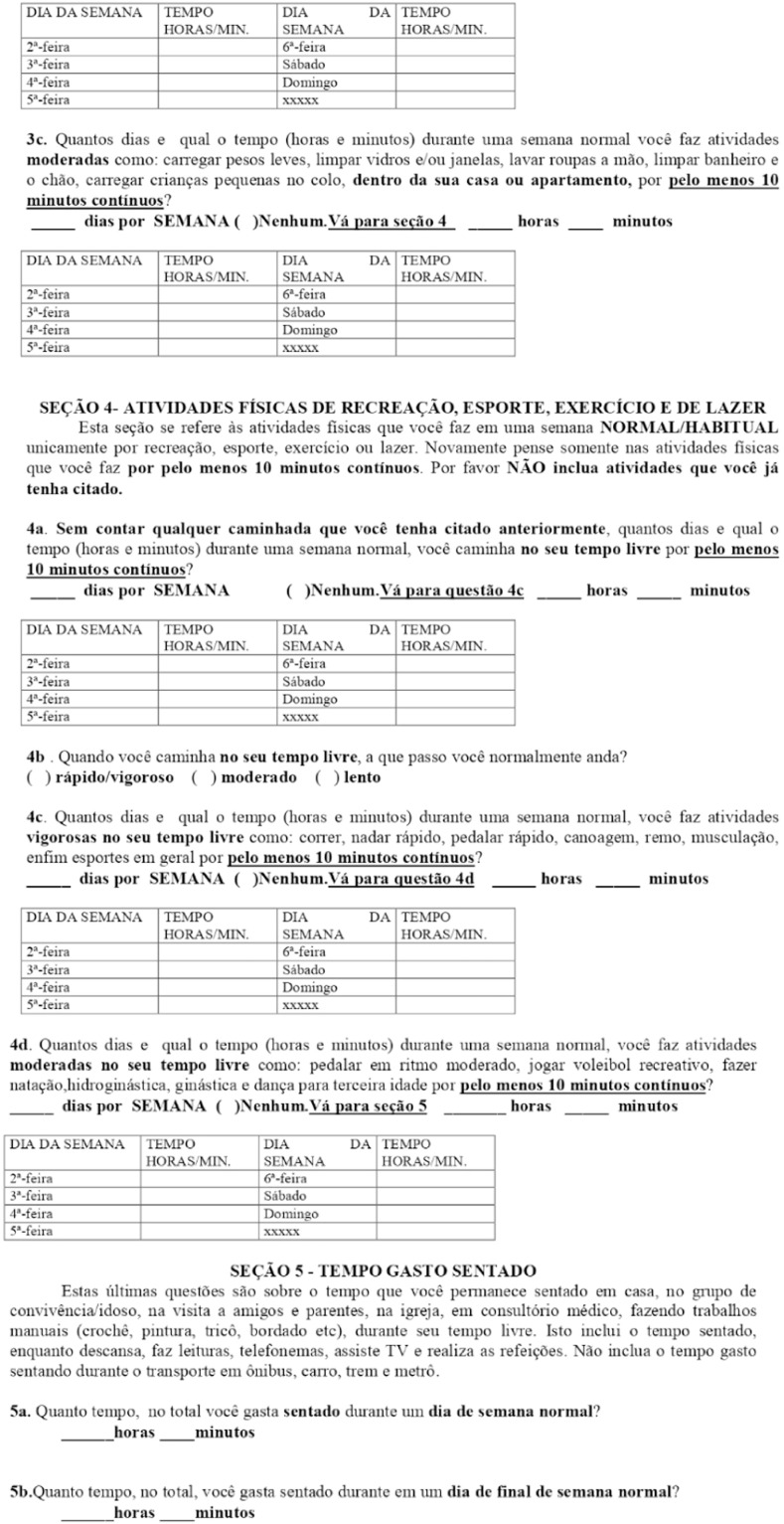
IPAQ- international physical activity questionnaire.

The International Physical Activity Questionnaire (IPAQ) is a five-section, standardized tool designed for quick and facilitated application. It assesses participants' habitual physical activity levels by evaluating the frequency and duration of physical activities performed in daily life (Fig. 2).

### Surface electromyography (sEMG)

Surface electromyography (sEMG) is a widely used research tool for quantifying neural control strategies that adapt to environmental and task-specific demands [6]. These strategies represent the signal output from the central nervous system (CNS) to execute movements via the peripheral nervous system (PNS) [6]. sEMG is a non-invasive technique that utilizes surface electrodes to measure muscle activity.

The amplitude of the sEMG signal is determined after an initial processing stage, which involves filtering and computing parameters such as the rectified mean square value, integrated value, or root mean square (RMS) from the raw data [6]. It is important to note that variations in electromyographic signal amplitude occur among individuals due to physiological and non-physiological factors, as well as detection variables that may influence data interpretation [6].

In this study, sEMG will be used to record motor potentials from the dominant lower limb’s quadriceps muscles, specifically the rectus femoris, vastus medialis, and vastus lateralis**.**

During data collection, participants will be positioned in a semi-recumbent posture, between sitting and supine. Electrode placement will follow the SENIAM (Surface Electromyography for the Non-Invasive Assessment of Muscles) guidelines, with the following anatomical references:

• **Rectus femoris**: Electrodes will be positioned at 50 % of the distance between the anterior superior iliac spine (ASIS) and the superior border of the patella.
• **Vastus medialis**: Electrodes will be positioned at 80 % of the distance between the ASIS and the joint space at the anterior edge of the medial ligament.
• **Vastus lateralis**: Electrodes will be positioned at ⅔ of the distance between the ASIS and the lateral border of the patella.

**Fig. 3:**
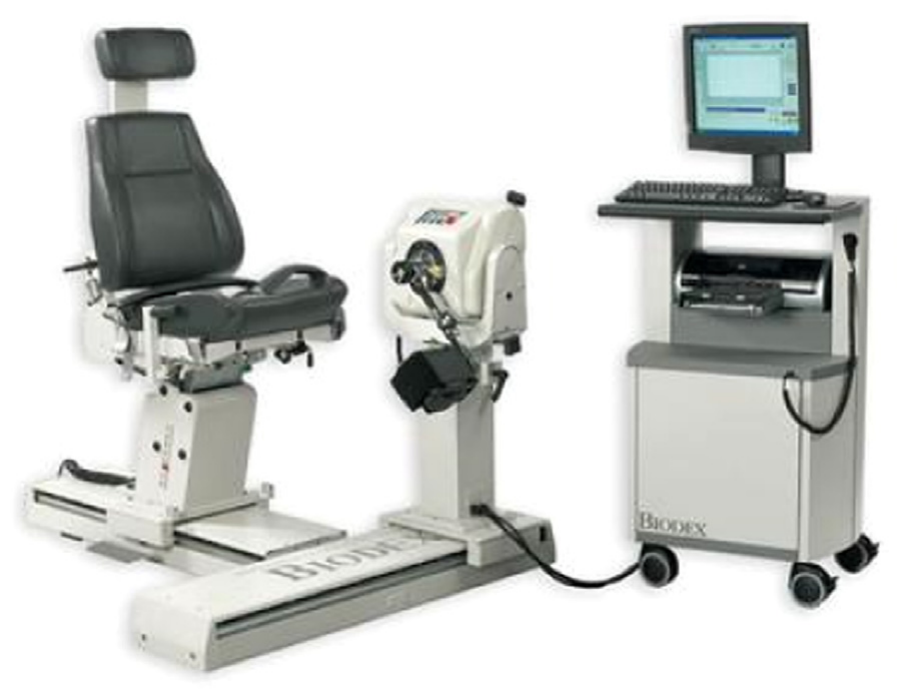
Isokinetic dynamometer- biodex.

**Fig. 4:**
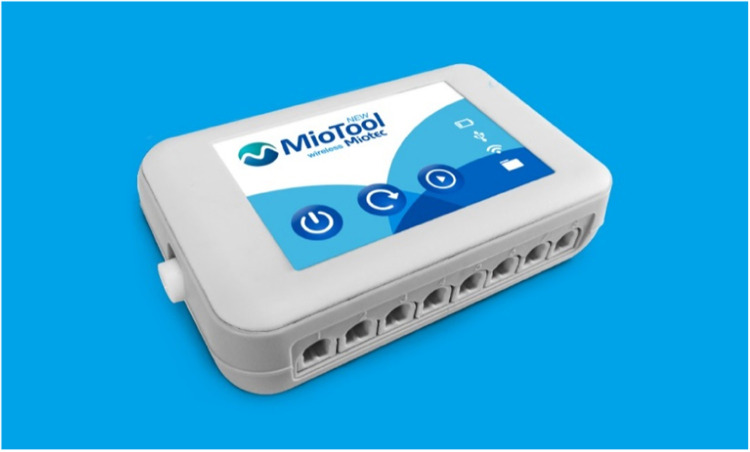
Surface Electromyography equipament miotool.

This standardized electrode placement ensures reproducibility and accuracy in sEMG measurements, contributing to the reliability of the study (Figs. 3 and 4).

### Protocol validation

The combination of non-invasive transcranial magnetic stimulation (TMS) with isokinetic dynamometry is expected to yield favorable results in assessing muscle performance parameters. This finding holds significant societal relevance, as positive outcomes could pave the way for further research aimed at refining this intervention for both healthy individuals and specific populations with distinct clinical needs.

## Limitations

The primary limitation of this study relates to the sample population, as it includes only male participants aged between 18 and 25 years. This restriction may lead to differences in outcomes if the study is replicated in populations with different characteristics, such as women, younger individuals, or older adults.

Furthermore, research on the effects of TMS on lower limb muscle strength remains scarce. The limited number of existing studies often fails to account for interindividual variability in responses to TMS, which may affect the generalizability of findings. Another major challenge is the lack of standardization in TMS protocols. Variations in stimulation parameters—such as intensity, frequency, and the number of sessions—can significantly impact results, complicating both interpretation and reproducibility across different populations and research settings.

Additionally, controlling all variables that influence TMS response poses a challenge. Factors such as participants’ baseline physical conditioning, pre-existing musculoskeletal injuries, or medication use can affect lower limb muscle strength and responsiveness to stimulation, potentially confounding study results.

Despite these limitations, this protocol remains both valid and necessary. The clinical hypothesis suggests that TMS may effectively enhance lower limb muscle strength, offering potential applications in rehabilitation and performance optimization. The findings of this study could contribute to the development of novel therapeutic strategies across various populations, emphasizing the importance of further investigation in this field.

## Credit author statement

**Thiago Conceição dos Santos:** Conceptualization, development, writing of the original preparation of the manuscript; **Rildo Tavares dos Santos:** Conceptualization, development, writing of the original preparation of the manuscript; **Hudson Renato de Paula Oliveira:** Isokinetic Dynamometer trainer, review; **Felipe Mendes Barcelos Angeli**: development ilustrations; **Emanuelly Augusta da Silva Bispo:** Translater; **Ian Manhoni Baiense:** TMS trainer, review; **Walter Gomes da Silva Filho:** Supervision, review and Original Draft Preparation; **Guilherme Peixoto Tinoco Areas:** Methods and Statistics; **Fernando Zanela da Silva Arêas:** Supervision, Manuscript Revision, and Editing.

## Disponibilidade de dados

None of the data described in the article has been used in research.

## Declaration of competing interest

The authors declare that they have no known competing financial interests or personal relationships that could have appeared to influence the work reported in this paper.

## Data Availability

Data will be made available on request.

## References

[1] Powers S., et al. Fisiologia do Exercício. 9ª ed. Manole. 2015.

[2] Guyton A.C., Hall M.E., Hall J.E. (2021).

[3] Jannati A., Oberman L.M., Rotenberg A., Pascual-Leone A. (2023). Assessing the mechanisms of brain plasticity by transcranial magnetic stimulation. Neuropsychopharmacology.

[4] Hirano M., Katoh M., Gomi M., Arai S. (2020). Validity and reliability of isometric knee extension muscle strength measurements using a belt-stabilized hand-held dynamometer: a comparison with the measurement using an isokinetic dynamometer in a sitting posture. J. Phys. Ther. Sci.

[5] Gilio F. (2002). Repetitive magnetic stimulation of cortical motor areas in Parkinson's disease: implications for the pathophysiology of cortical function. Mov. Disord..

[6] Kambič T., Lainščak M., Hadžić V. (2020). Reproducibility of isokinetic knee testing using the novel isokinetic SMM iMoment dynamometer. PLoS One.

